# Identifying Type 2 Diabetic Brains by Investigating Disease-Related Structural Changes in Magnetic Resonance Imaging

**DOI:** 10.3389/fnins.2021.728874

**Published:** 2021-10-26

**Authors:** Yuna Chen, Yongsheng Pan, Shangyu Kang, Junshen Lu, Xin Tan, Yi Liang, Wenjiao Lyu, Yifan Li, Haoming Huang, Chunhong Qin, Zhangzhi Zhu, Saimei Li, Shijun Qiu

**Affiliations:** ^1^Department of Endocrinology, The First Affiliated Hospital of Guangzhou University of Chinese Medicine, Guangzhou, China; ^2^Postdoctoral Research Station, Guangzhou University of Chinese Medicine, Guangzhou, China; ^3^School of Biomedical Engineering, ShanghaiTech University, Shanghai, China; ^4^School of Computer Science and Engineering, Northwestern Polytechnical University, Xi’an, China; ^5^Department of Radiology, The First Affiliated Hospital of Guangzhou University of Chinese Medicine, Guangzhou, China; ^6^Guangxi School of Traditional Chinese Medicine, Nanning, China

**Keywords:** type 2 diabetes mellitus, cognitive impairment, machine learning, medical image, structural MRI

## Abstract

Diabetes with high blood glucose levels may damage the brain nerves and thus increase the risk of dementia. Previous studies have shown that dementia can be reflected in altered brain structure, facilitating computer-aided diagnosis of brain diseases based on structural magnetic resonance imaging (MRI). However, type 2 diabetes mellitus (T2DM)-mediated changes in the brain structures have not yet been studied, and only a few studies have focused on the use of brain MRI for automated diagnosis of T2DM. Hence, identifying MRI biomarkers is essential to evaluate the association between changes in brain structure and T2DM as well as cognitive impairment (CI). The present study aims to investigate four methods to extract features from MRI, characterize imaging biomarkers, as well as identify subjects with T2DM and CI.

## Introduction

As a general disease with high blood glucose, diabetes is the seventh leading cause of disability worldwide (Novoselova et al., 2014; Khan et al., 2020). Glucose requires the help of insulin, produced by the pancreas, to enter into the cells and be used for energy. If insulin is not sufficient or not well-used, glucose will remain in the blood and not reach the cells. Health problems may arise with excessive glucose in the blood over time. Typically, type 2 diabetes mellitus (T2DM) is the most common type of diabetes and accounts for 90–95% of diabetes cases.

As a heterogeneous disorder characterized by insulin resistance and hyperglycemia, T2DM affected 463 million people worldwide in 2017 (Karamzad et al., 2020). According to the Diabetes Atlas 7th Edition, T2DM is expected to reach 642 million by 2040 (Atlas et al., 2015). T2DM patients may undergo a gradual progression from normal glucose metabolism to impaired glucose metabolism and become T2DM. Several studies have shown that T2DM with high blood glucose level over a long period may damage the brain nerves and thus increase the risk of cognitive impairment (CI) and dementia (Vieira et al., 2018; Biessels and Whitmer, 2020; Sharma et al., 2020; Srikanth et al., 2020). Currently, the occurrence of CI is an irreversible process and has little effective cure. Thus, preventive treatments are needed in the early stages of dementia. An accurate and objective method to help to identify T2DM and CI is an urgent requirement.

Brain imaging is a vital tool for exploring the mechanisms linking T2DM and CI. Non-invasive structural magnetic resonance imaging (MRI) is one of the most common brain imaging modalities used in brain research. Scattered reports have demonstrated that glucose alterations are related to regional brain changes and CI. Some studies favor the hypothesis that the brain structural changes in T2DM encompass both gray matter (GM) and white matter (WM) alterations (Mankovsky et al., 2018). Other studies revealed that patients with T2DM had CI and structural brain alterations (Biessels and Despa, 2018), such as in the hippocampus (Gold et al., 2007; Li et al., 2020). In addition, patients with T2DM showed brain atrophy, including low total and regional white and gray matter volumes (Moran et al., 2013; Garc´ıa-Casares et al., 2014; Mehta et al., 2014; Wu et al., 2017). Furthermore, the volume of white matter hyperintensities also appeared to be modestly increased in people with T2DM rather than those without T2DM (Moran et al., 2017). These brain abnormalities might be potential imaging biomarkers for T2DM or T2DM with cognitive impairment (T2DM-CI). Some reports discovered that the topological structure of the white matter network was altered in T2DM patients, and this abnormal network structure was related to the executive dysfunction (Zhang J. et al., 2011), while others mentioned that T2DM disturbed the overall topological features of gray matter networks (Cao et al., 2019). However, the pathophysiological mechanisms underlying T2DM or T2DM-CI are yet to be clarified.

Machine learning methods are utilized to detect brain activity in neuroimaging and assess brain structure or function to discriminate between groups or conditions. It would be interesting to understand whether machine learning methods can differentiate patients from healthy controls using MRI. Our previous study performed classification tasks between T2DM-CI and healthy controls (HC), as well as T2DM without cognitive impairment (T2DM-noCI) and HC using a high-order brain network construction method (dHOFC) (Chen et al., 2021). A few studies also utilized the machine learning method as the classifier for the diagnosis of diabetes. Pima Indians Diabetes Database (PIDD), including personal data (age and number of times pregnant) and results of medical examination (blood pressure, body mass index, and plasma glucose concentration), was used for diagnosis. El-Baz et al. (2016) used committees of neural network-based classifiers to detect T2DM using combined multi-layer perceptron and combined cascade-forward backpropagation network classifiers. Yue et al. (2008) combined Quantum Particle Swarm Optimization and weighted least squares support vector machine (SVM) to diagnose T2DM. However, limited classification is available using structure-based machine learning to identify T2DM from individuals without T2DM (NT) and CI from people with normal cognition (NC).

Therefore, we utilized four feature extraction methods, including a volume-of-regions (VOR) method, a patch-based morphometry (PBM) method, a local energy pattern (LEP) method, and a deep transfer learning (DTL) method based on a pre-trained network (Pan et al., 2019a) to characterize the imaging biomarkers in T2DM and CI. Also, it is imperative to study computer-aided T2DM and CI diagnosis based on brain structure.

## Materials and Methods

### Participants

A total of 123 subjects (with 58 T2DM subjects and 65 NT subjects) from October 2018 to December 2020. This study was approved by the Ethics Committee of The First Affiliated Hospital of Guangzhou University of Chinese Medicine, Guangzhou, China. All participants provided written informed consent. Among the T2DM subjects, 25 subjects had CI, and the remaining 33 subjects had NC. Among the NT subjects, 15 had CI and 50 had NC. CI was defined as either T2DM or NT subjects with a Montreal Cognitive Assessment (MoCA) score <26 (Nasreddine et al., 2005). NC was defined as a T2DM or NT subject with a MoCA score ≥26. The T2DM patients were diagnosed by experienced endocrinologists at The First Affiliated Hospital of Guangzhou University of Chinese Medicine. The diagnostic criterion was either fasting plasma glucose (FPG) level ≥7.0 mmol/L or 2-h oral glucose tolerance test (OGTT) glucose level ≥11.1 mmol/L (Association, 2014). Participants with severe head injury, intracranial organic diseases (such as tumors, infections, cerebrovascular accidents, congenital brain dysplasia, and obvious variations), brain surgery, positive neurological symptoms, alcohol abuse, level-3 hypertension, or heart attack were excluded from the study.

### Magnetic Resonance Image Acquisition

All participants underwent scanning on a 3.0-T GE scanner (SIGNA EXCITE GE Medical Systems, United States) with an 8-channel head coil at the imaging department of The First Affiliated Hospital of Guangzhou University of Chinese Medicine. The scan time was within 1 week after enrollment. Structural images were acquired using a three-dimensional (3D) magnetization-prepared rapid-acquisition gradient echo sequence with the following parameters: repetition time (TR) = 8.15 ms, echo time (TE) = 3.17 ms, flip angle = 12°, slice thickness = 1 mm, slice gap = 0 mm, number of excitations (NEX) = 1, field of view (FOV) = 256 mm × 256 mm, matrix size = 256 × 256, sagittal slices = 188, and total scanning time = 250s. To minimize head movement, we used foam pads to fix the head before scanning. Participants were asked to lie in a supine position, keep their eyes closed, not fall asleep, and avoid ideological activities. Two experienced radiologists monitored the image quality and participants during the scan and terminated the acquisition if the images were abnormal or participants were uncomfortable.

### Image Processing

**FIGURE 1:**
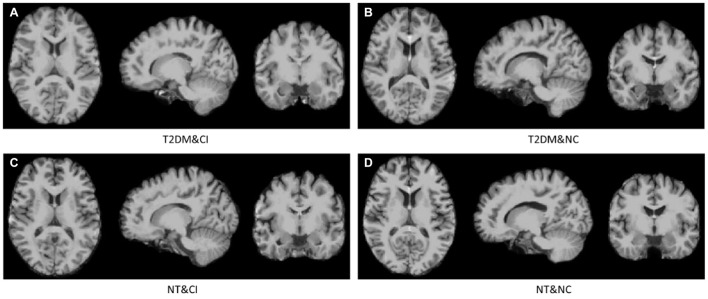
Four cases in our dataset. From **(A–D)** are a T2DM with CI (T2DM&CI) subject, a T2DM with NC (T2DM&NC) subject, a NT with CI (NT&CI) subject, and a NT with NC (NT&NC) subject.

All magnetic resonance (MR) images were first subjected to a Non-parametric Non-uniform intensity Normalization (N3) correction, and then pre-processed by Statistical Parametric Mapping (SPM) toolbox using MATLAB in three steps as follows: (1) Each brain volume was segmented into GM, WM, cerebrospinal fluid (CSF), skull, scalp, and background; (2) The GM, WM, and CSF were conjuncted with a hole-filling morphological operation to create a brain mask that would facilitate the classification algorithm to focus on the brain region; (3) Each volume was spatially normalized to the Montreal Neurological Institute (MNI) space using SPM. After pre-processing, all the brain MR images were found to have the same size (181 × 217 × 181) as the MNI template. Four cases with similar education years are visualized in Figure 1: (a) a T2DM with CI subject, (b) a T2DM with NC subject, (c) a NT with CI subject, and (d) a NT with NC subject. These methods were conducted on processed data for optimal comparison, i.e., all features were extracted from the normalized space.

### Method

MRI biomarkers measure the difference between T2DM subjects and NT subjects, and hence, should be identified. Thus, the present study aimed to investigate the effectiveness of four feature extraction methods, including (1) a VOR method, (2) a PBM method, (3) a LEP method, and (4) a DTL method, to characterize the imaging biomarkers in brain MRI data. The feature dimensions of VOR, PBM, LEP, and DTL were 116, 864, 93,312, and 7,680, respectively. A general flowchart of the initially extracted features is shown in Figure 1.

(1) VOR first extracts the volume of GM tissue in 116 regions of interest (ROIs) defined by the anatomical automatic labeling (AAL) atlas and then normalizes to the total GM volume of each brain. Based on previous studies (Walhovd et al., 2010; Cuingnet et al., 2011; Zhang D. et al., 2011) we extracted the VOR features that were first aligned to the AAL atlas (Tzourio-Mazoyer et al., 2002) to the native space of each subject using SPM12 (Friston et al., 2007). Then, the GM inside those ROIs in each MRI scan was extracted and normalized to the total GM volume of each brain, followed by a linear SVM using default parameters (i.e., *C* = 1) for brain disease classification.

(2) PBM first crops each brain image to 144 × 192 × 128 to remove the non-brain regions and the partitions it to 9×12×8 patches with size of 16 × 16 × 16. Then, the volumes of GM tissue were extracted from those patches, followed by normalization to the total GM volume. Following the protocols of Liu et al. (2012, 2014), the PBM method partitions each brain image into small 3D patches and combines the features extracted from selected patches at the classifier level. In this work, each brain image is partitioned into multiple 16 × 16 × 16 patches, and the feature representation of each brain is extracted from these patches. Next, we extracted the volumes of GM tissue inside these patches from MRI scans similar to the ROI representation. The dimension of the extracted feature is 864, where each dimension corresponds to a patch. The MRI-based features are normalized by the total volume and fed to a linear SVM for classification.

(3) LEP extracts the local energy patterns from a several locations centered at grid patches (size is 16 × 16 × 16) and then concatenates these features from multiple locations to represent each brain. This method extracts the local energy patterns (Zhang et al., 2013), which represent the response of sever filters, from a local patch centered at each grid patch. The majority of the local energy patterns are histogram-like statistical features of local patches, such as the local binary pattern, Gabor filters, Histograms of Oriented Gradients (HOG), and other steerable filters. In this study, we directly used HOG to represent a local patch. Since the patches from the brain images are 3D, we calculated HOG in each slice and each of the three views, i.e., the axial, coronal, and sagittal views, and considered the average of the features of each patch. Then, these features from multiple patches were concatenated, followed by a z-score normalization (Jain et al., 2005) process. As the dimension of HOG is 36, the total dimension of the LEP representation of an image is 36×3×864 = 93312. Finally, the derived features represent each subject, followed by a linear SVM model for classification.

(4) DTL applies a convolutional neural network (CNN) to our collection and extracts the feature map of the last convolutional layer to represent each image. Pan et al. (2019a) used CNN to employ the structural parameters of the diagnosis model that were pre-trained on the ADNI1 dataset with baseline MRI of 845 subjects. These ADNI images were also subjected to N3 correction similar to our study, which aligned the processed ADNI and our images to have same intensity range. As the output of the feature extraction part of this pre-trained CNN was 5×6×4×64, the dimension of the final DTL feature of an image was 7,680. Finally, the derived DTL features of each MRI scan were used to represent each subject, followed by the linear SVM model for classification.

Herein, we used a cheap calibration technique to address poor probability estimation from SVMs, as described previously (Lin et al., 2017; Pan et al., 2019b). The cheap calibration technique maps the predicted output in an injective monotonic manner, facilitating maximum gain of SVMs (with margin maximizing) from calibration. The evaluation metrics were unaffected by calibration, while the probability metrics were improved. Therefore, we used a cheap calibration technique rather than manually selected optimal parameters for SVMs.

## Experiments and Results

### Information and Comparison of Clinical Characteristics

The information and comparison of clinical data of the 123 subjects are summarized in Table 1, and the characteristics showing significant group differences (*P* < 0.05) were marked with *. Statistical comparisons between T2DM and NT did not reveal any significant differences in age, gender, or education level, while the statistical comparisons between CI and NC showed no significant differences in age. However, some clinical characteristics such as MoCA scores indicated significant differences between T2DM and NT, as well as between CI and NC. Gender and education level also exhibited significant differences between CI and NC.

**TABLE 1:** Comparison of clinical characteristics between two groups.

	T2DM (*n* = 58)	NT (*n* = 65)	*P* value	CI (*n* = 40)	NC (*n* = 83)	*P* value
Age (years)	51.17 ± 9.28	48.34 ± 7.39	0.062	51.00 ± 9.15	49.04 ± 8.03	0.23
Gender (M/F)	34/24	31/34	0.28	15/25	50/33	0.021*
Education (years)	9.83 ± 4.32	9.94 ± 3.90	0.88	7.30 (0, 15)	11.13 (2, 19)	0.0001*
MoCA	25.12 (16, 30)	26.75 (20, 30)	0.016*	22.43 (16, 25)	27.70 (26, 30)	0.0001*

*T2DM, type 2 diabetes mellitus; NT, individuals without T2DM; CI, cognitive impairment; NC, normal cognition; M, male; F, female; MoCA, Montreal cognitive assessment.*

The characteristics showing significant group differences (*P* < 0.05) were marked with *.

### Classification Performance

We performed experiments on two tasks: (1) distinguishing CI subjects from NC subjects and (2) distinguishing T2DM subjects from NT subjects. A leave-one-out cross-validation strategy was applied to evaluate the performance of four feature representations. Specifically, in each fold, one subject was used for test data, while the remaining were used for training data to learn a linear SVM classifier (with the default parameter *C* = 1). Six metrics were used for performance evaluation, including accuracy (ACC), sensitivity (SEN), specificity (SPE), F1-Score (F1S), receiver operating characteristic (ROC) curve, and area under the ROC curve (AUC).

**FIGURE 2:**
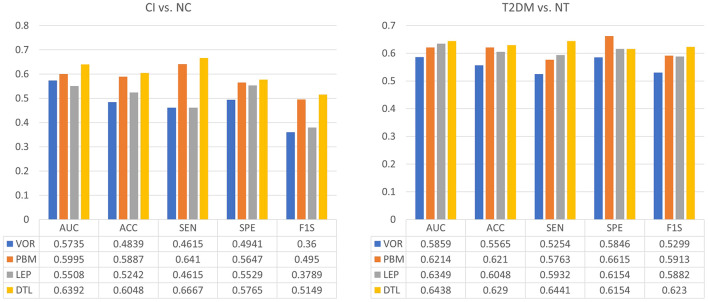
Performance of four different feature extraction methods in both the tasks of discriminating cognitive impairment (CI) subjects from normal cognition (NC) subjects (left), as well as type 2 diabetes mellitus (T2DM) subjects from individuals without T2DM (NT) (right).

**FIGURE 3:**
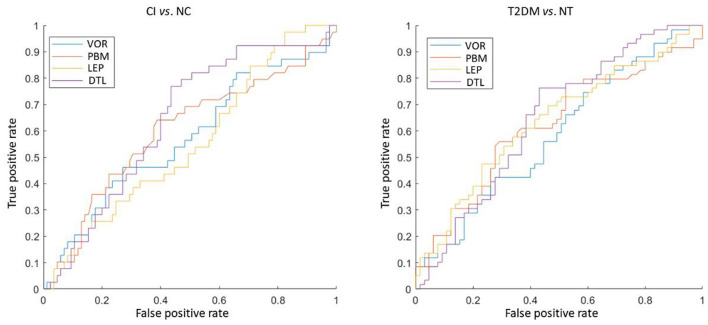
The ROC curve of four different feature extraction methods in both discriminating the cognitive impairment (CI) subjects from normal cognition (NC) subjects (left), as well as type 2 diabetes mellitus (T2DM) subjects from individuals without T2DM (NT) (right).

The classification results of four methods in two tasks were shown in Figure 2. We observed that DTL achieves the best performance in both CI vs. NC, as well as T2DM vs. NT classification tasks. The ROC curves of these methods were also shown in Figure 3, indicating that DTL achieves a higher true-positive rate compared to other methods. This phenomenon suggested that DTL can detect the abnormality of brain structure. In addition, the best AUC values achieved by DTL for CI vs. NC and T2DM vs. NT classification are 0.6438 and 0.6392, respectively, which are not promising. This could be attributed to DTL training and that it is applied on two datasets with different data distributions (i.e., with MRIs acquired from Asian and American brains, respectively). Interestingly, data adaptation/harmonization is essential to boost identification performance.

We further selected CI subjects to classify T2DM vs. NT, the AUC values are 0.5857, 0.6286, 0.5771, and 0.6186, respectively, for VOR, PBM, LEP, and DTL. Such results are similar to that with using all subjects and have verified the challenge to classify T2DM vs. NT. More convincing results should be achieved in our future work by collecting more data and redoing the experiments.

## Discussion

To the best of our knowledge, this is the first classification study between T2DM and NT, as well as CI and NC based on brain structure using four feature extraction methods. The results indicated that DTL achieves the best performance in both CI vs. NC and T2DM vs. NT classification tasks. As neural network generally needs a large training dataset, it may not be stable to train a neural network from scratch. As an alternative, pre-train in a large dataset to extract features is a good choice. As ADNI is the largest dataset for cognition study, it may be the most suitable choice to train a network to extract features on our data. Meanwhile, using the features extracted by this pre-trained network can outperform the other handcraft features, which verified the advantage of pre-trained network.

Nevertheless, the present study has several limitations that should be considered during the interpretation of the findings. First, our sample size is relatively small. Thus, enrolling more participants to perform machine learning in the future is essential. As most of our features are high-dimensional, it may not be suitable to use non-linear SVM. We have tried non-linear SVM, the results may be a little higher if selected good parameters. However, since this may hurt the objectivity, we did not report the results of non-linear SVM. As we follow a unique pre-processing, we thought this may not be the issue. The reason for the low performance may lie into the data content, for which, we will collect more data to verify that in our future work. Second, a significant difference was observed between T2DM and NT in MoCA scores. The classification results might be affected by subjects with CI. Third, we did not match gender or education level between CI and NC subjects. The education level might influence cognitive ability. Therefore, in future studies, we would consider subjects with different education levels to avoid putative bias.

## Conclusion

This study investigated four feature extraction methods to characterize the potential abnormalities in the brain structure caused by T2DM in brain MRI data. Experimental results indicate that DTL is possible to achieve a better performance than others, which helps to analyze CI caused by T2DM.

## Data Availability Statement

The original contributions presented in the study are included in the article/supplementary material, further inquiries can be directed to the corresponding authors.

## Ethics Statement

The studies involving human participants were reviewed and approved by the Ethics Committee of The First Affiliated Hospital of Guangzhou University of Chinese Medicine, Guangzhou, China. The patients/participants provided their written informed consent to participate in this study. Written informed consent was obtained from the individual(s) for the publication of any potentially identifiable images or data included in this article.

## Author Contributions

SQ, SL, and ZZ proposed the study concept and designed the experiments. YC and YP wrote the manuscript. XT, YLia, and SK collected the data of MRI and retrieved the relevant literature and materials. YLi and CQ performed the experiments. JL, WL, and HH constructed the figures and tables. All authors contributed to the article and approved the submitted version.

## Conflict of Interest

The authors declare that the research was conducted in the absence of any commercial or financial relationships that could be construed as a potential conflict of interest.

## Publisher’s Note

All claims expressed in this article are solely those of the authors and do not necessarily represent those of their affiliated organizations, or those of the publisher, the editors and the reviewers. Any product that may be evaluated in this article, or claim that may be made by its manufacturer, is not guaranteed or endorsed by the publisher.
